# Simulated Usability Testing: A Modified Approach to Healthcare Research in Prison

**DOI:** 10.1093/geroni/igaf122.1034

**Published:** 2025-12-31

**Authors:** Erin Kitt-Lewis, Susan Loeb, Brandon Herbeck, Barbara Walkosz

**Affiliations:** Penn State, State College, Pennsylvania, United States; The Pennsylvania State University, University Park, Pennsylvania, United States; Klein Buendel, Golden, Colorado, United States; Klein Buendel, Golden, Colorado, United States

## Abstract

Changes in research policies and competing priorities contribute to extended research review periods and in many cases denial of access to prisons for health-related research. To gain access, investigators must approach challenges and restrictions with an openness to modified approaches to their original study plans, while simultaneously honoring their study’s aims. The purpose of this presentation is to describe a pragmatic approach to usability testing Just Care, an electronic learning program designed to prepare vetted people living in prison to assist staff in care for those needing geriatric or end-of-life care. Several Department of Corrections (DOCs) either discouraged our researchers from applying or denied the application with the original large scale usability testing plan. Researchers persevered and one DOC was willing to discuss the potential for a modified application. Collaborative brainstorming between researchers and the DOC resulted in a modified approach to present to the DOC review board. This was termed, simulated usability testing and included paper documents; participants viewing the e-learning program remotely as part of a focus group (FG) and followed by one FG discussion with staff (n = 4 at each of two prisons) and two FGs with people living in prison (n = 16 at each of two prisons). Seizing opportunities to collaborate with community partners to create pragmatic solutions to safety, security, and burden-related constraints in prisons can make accessing prison populations a reality. Gaining access while honoring the original study aims is critical to continuing supporting evidence-based solutions for those living in prison.

